# Evaluation of the quality of depression-related information on Chinese websites and video platforms: a cross-sectional comparative analysis

**DOI:** 10.3389/fpsyt.2024.1408384

**Published:** 2024-12-12

**Authors:** YueDong Chen, Jia Yin, YuKe Ding, ChangYu Wang, JiaXin Zhu, Lu Niu

**Affiliations:** Department of Social Medicine and Health Management, Xiangya School of Public Health, Central South University, Changsha, China

**Keywords:** information quality, internet, depression, Chinese websites, Chinese videos

## Abstract

**Objectives:**

We aimed to assess the quality of information regarding depression on Chinese websites and popular video platforms.

**Methods:**

We conducted searches on website platforms (Baidu, Bing) and video platforms (Bilibili, Douyin) using search terms “depression”, “depressive disorder”, “depression treatment”, “depressive anxiety”, “depressed patient”, and “depressive symptoms”. We collected the first 50 results with each search term in each platform. Each website and video included in this study was assessed using The DISCERN instrument (DISCERN), Journal of American Medical Association benchmark criteria (JAMA), Hexagonal Radar Schema (HRS), and Global Quality Scores (GQS).

**Results:**

A total of 177 websites, 216 Bilibili videos, and 244 Douyin videos were included. Among all the platforms, websites had the highest median scores on DISCERN and HRS, at 33 and 2 respectively, but were still classified as “poor” and “very poor” according to the classification. Bilibili, Douyin, and websites had median scores of 3, 2, and 2 respectively in JAMA, indicating a moderate level of quality. Bilibili, Douyin, and websites all had a median score of 2 for GQS, and were of poor quality. Only the percentage score for JAMA was more than half of the weighted score, while none of the other scales reached half of the score. The median percentage scores of the websites in DISCERN, HRS, and GQS were higher than those of Bilibili and Douyin (*P* < 0.001). Compared to other sources, news media on websites and medical organizations on videos demonstrated higher quality (all *P* values < 0.05).

**Conclusions:**

The findings of the study indicated an overall low quality of online depression information. Collaborative efforts between platforms and professionals are necessary to improve the comprehensiveness and quality of depression-related information available online. In addition, the platform needs to prioritize optimizing the algorithm of recommendations and present real high-quality health information to the audience.

## 1 Introduction

Depressive disorders have become a neglected global health crisis, with a particularly high prevalence in the youth population (1). Previous studies have found that depressive disorders are ranked 13th globally and 11th in China in terms of disease burden (2). The current 12-month prevalence of depressive disorders in China is 3.6% (3). Over the past three decades, there has been a 9.01% increase in the prevalence of depressive disorders in China (4). Depressive disorders not only impose a great economic burden on patients, but also cause many social and health problems, such as impairment of social functioning, reduced quality of life, unemployment, and even suicide, among other serious consequences (5, 6). Despite the profound negative effects of depressive disorders on patients’ quality of life, a mere 0.5% of affected individuals in China receive adequate treatment (3). The scarcity of treatment stems from a multifaceted array of factors, including restricted access to healthcare services, inadequate transportation, the misunderstanding and stigmatization surrounding mental health, and the financial costs of professional care (7–9). Patients often struggle to cope with the illness alone due to a lack of knowledge about the basics of depression, and it is difficult for them to cope with the condition independently (10).

The Internet’s rapid growth has expanded access to health information and medical resources related to depression (11). Since China proposed the “Healthy China” strategy in 2016, public awareness of Internet healthcare has grown (12). By December 2023, China’s Internet user base reached 1.092 billion, with a penetration rate of 77.5% (13). People are increasingly shifting from traditional search engines like Google, Baidu, and Bing to decentralized, community-driven searches on social platforms such as WeChat, RED, and Douyin, particularly for video-based content that delivers information intuitively and engagingly (13, 14). However, the extensive availability of online depression-related information is a double-edged sword: while it offers convenient, private, and comprehensive resources that support healthy behaviors, the variable quality of information can lead to negative effects (15, 16). The rapid dissemination of user-generated content, often lacking quality control, has led to increased anxiety and misinformation, impacting people’s health management and behaviors (17). With the rising number of individuals affected by depressive disorders, the demand for online health information on depression has grown significantly (18). Guided by the “Healthy China” strategy, there is an urgent need to assess the quality of depression-related information available online, to clarify the reliability of popular content on digital platforms, and to ensure the public has access to appropriate health services and information resources.

Previous studies have assessed the quality of websites containing depression-related health information through search engines, using tools such as the DISCERN instrument (DISCERN), the Patient Education Materials Assessment Tool, and Health on the Net (19–21). These studies generally found that most websites were of low quality, although some highlighted high-quality content on select sites (19, 22). Research on specific types of depression-related information, such as Perinatal Depression and Late-Life Depression, also indicated that website quality could be improved (11, 23). With the rise of social media, video content has become increasingly popular, and studies evaluating health topics like Testicular Torsion and Gallstone Disease on video platforms have shown substandard quality (24, 25). Since these studies typically focus on single platforms, however, it remains unclear whether quality differences exist across different platform types. One study compared the quality of health information on Cosmetic Injectables across websites and video platforms, finding that video content was generally of lower quality than website content (26). Currently, no studies have compared the quality of depression-related health information across platform types, nor have any studies specifically evaluated this information on Chinese Internet platforms. To address this research gap and based on previous findings, this study proposes the following hypotheses:

1. The overall quality of depression-related health information on Internet platforms is low.
2. The quality of depression-related health information is higher on search engine platforms than on video platforms.

In response to these concerns, this study aims to evaluate and compare the quality of the internet as an information source for depression by utilizing popular Chinese search engines and video platforms.

## 2 Methods

### 2.1 Data collection

The two most popular search engines and video platforms in mainland China were selected, namely Baidu and Bing, as well as Bilibili and Douyin, respectively. These selections were based on the overall Traffic Ranking from the Similar web website (27). The search terms “抑郁” (depression), “抑郁症” (depressive disorder), “抑郁治疗” (depression treatment), “抑郁焦虑” (depressive anxiety), “抑郁症患者” (depressed patient), and “抑郁症状” (depressive symptoms) were selected based on their common usage by Chinese users seeking depression-related information (28). Previous studies have found that users typically do not browse beyond the first three pages of results and often analyze data from the top 30 or 50 items. To improve comparability between videos and websites and reduce selection bias, we collected data from the top 50 videos and websites retrieved for each search term (26, 29). To minimize any potential impact on search results and outcomes, we implemented several procedures. This included the registration of new accounts for video platforms, clearing search history and cookies in search engines, and reinstalling video software. These steps were taken to ensure a fresh search environment and reduce any potential bias in the results. The study data are all open access public data and therefore do not require ethics committee approval.

To minimize bias from factors like manipulation and equipment, data search and collection were conducted independently by a single researcher using specialized equipment in Changsha, Hunan, China, on April 12, 2023. Various website features were recorded, including platform name, search term, website link, website title, search order, author, upload date, and website visits. For videos, we recorded the platform name, search term, video link, title, video author, number of likes, comments, shares, favorites, upload date, duration, and whether the identity was officially certified. The popularity of a video can be assessed through various engagement metrics, including the number of likes, comments, shares, and favorites. To comprehensively evaluate video popularity, we employed the Video Power Index (VPI), calculated as follows: (0.25 × likes + 0.25 × comments + 0.25 × favorites + 0.25 × shares)/(likes +comments + favorites + shares) ×100% (30). Since we did not have access to the number of views on Douyin, we adjusted this calculation method to obtain more comprehensive data.

### 2.2 Data cleaning and classification

**Figure 1:**
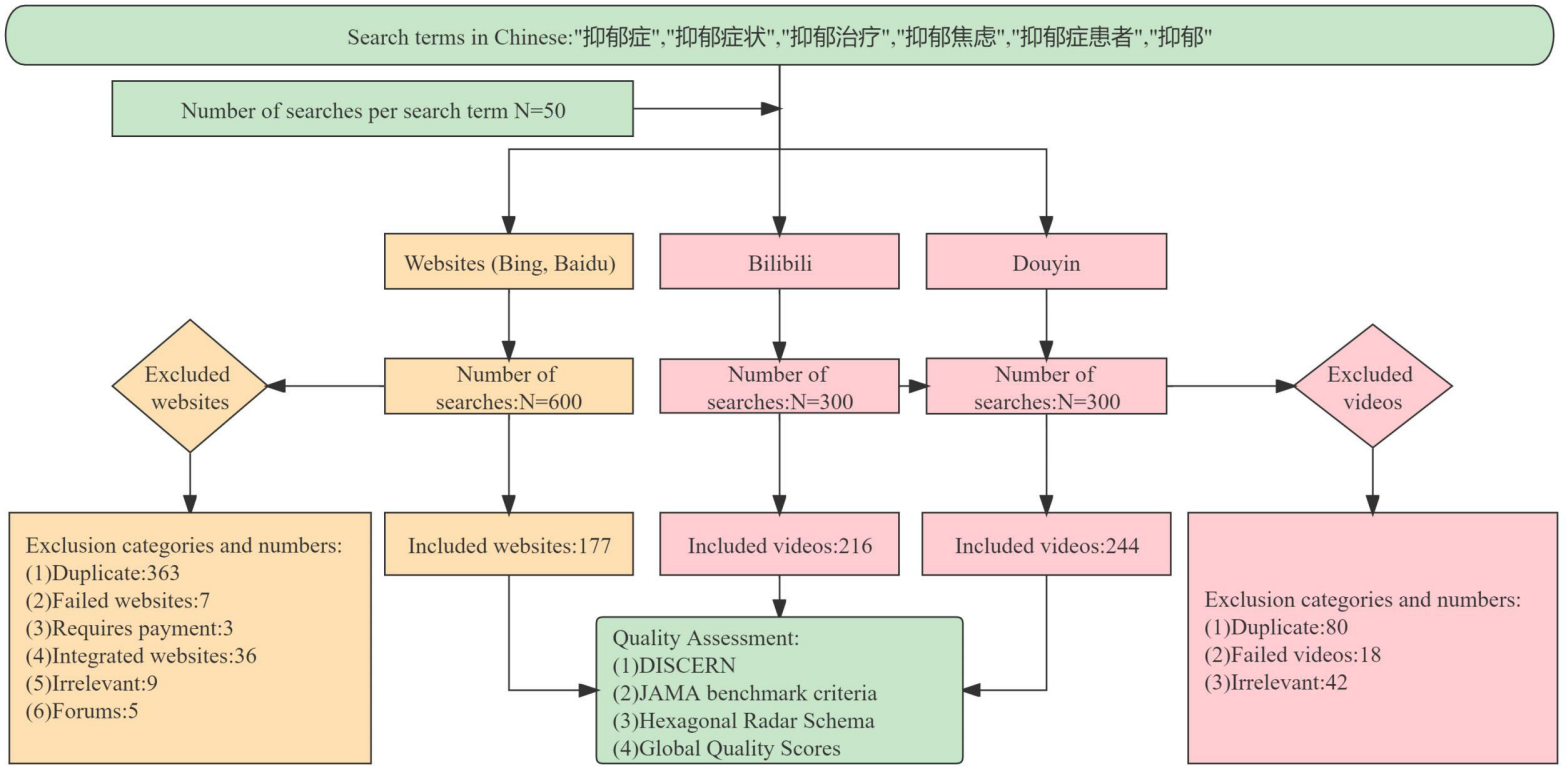
Flowchart of the selection of websites and videos for analysis.

Duplicate websites and videos with the same titles or URLs were automatically removed using Python (version 3.10.2). A manual content check was then conducted to further exclude ineligible websites and videos. The eligible websites and videos were reconfirmed by the first reviewer (**Figure 1**). To facilitate classification, we categorized the sources of websites and videos as medical websites, government websites, commercial websites, news media websites, and other websites, based on attributes and statements. The sources of videos were classified as doctors, non-medical professionals, news media, and medical organizations, based on identification status on the platforms. The content of both websites and videos was classified into disease symptoms, disease diagnosis, disease management, and disease risk factors, based on the characteristics of the disease content.

### 2.3 Evaluation of data

The DISCERN, Journal of American Medical Association benchmark criteria (JAMA), Hexagonal Radar Schema (HRS), and Global Quality Scores (GQS) were used to evaluate the quality of websites and videos. Specialized medical issues related to depression were referenced from the *Chinese Guidelines for the Prevention and Treatment of Depressive Disorders* (2nd edition) (31).

The DISCERN was initially adopted to assess the quality of publications but is now widely used to evaluate the quality of health-related information on the internet. Comprising three sections encompassing a total of 16 question in 3 sections, each question is assigned a score ranging from 1 to 5. The first section assesses the reliability of information (questions 1-8), the second section is to evaluate the quality of treatment information provided by the source (questions 9-15), and the third section is to assess the overall quality of objects based on the above quality (question 16). The DISCERN scoring criteria classified scores as follows: very poor (<27), poor (27-36), fair (39-50), good (51-62), and excellent (63-75) (32).

The JAMA were used to assess the reliability of online information sources, including four indexes: authorship, attribution, disclosure, and currency. Each index met the criterion counted as 1 point, with a maximal score of 4 points for all (33).

The HRS is a coded scale to reflect health information’s six dimensions, consisting of the definition, signs, risk factors, examinations, management, and disease outcomes, which provide a comprehensive framework to evaluate the quality of video or website-specific content. Each dimension score from 0 (Not addressed at all) to 2 (Fully addressed). Total scores were calculated and categorized into five grades using the original scale: very poor (<2.4), poor (2.4-4.8), fair (4.8-7.2), good (7.2-9.6), and excellent (9.6-12). By visually presenting the shape and size of a radar chart, the overall scores for different objects can be weighted and compared (34).

The GQS is based on the quality, flow, and usability to assess its overall score of information source, ranging from 1 (Poor quality; the poor flow of the site; most information missing; not at all) to 5 (Excellent quality and excellent flow; very useful for patients) levels (35).

Detailed information about the DISCERN, JAMA, HRS, and GQS can be found in **Supplementary Materials** (**Supplementary Tables 1**–**4**).

Two reviewers conducted the evaluation independently. Before the formal assessment, each sample was individually coded, and an evaluation form was created based on the selected tools. Three clinical psychiatrists and the two reviewers initially evaluated a subset of samples independently, with an Intraclass Correlation Coefficient (ICC) of 0.982. Psychiatrists then discussed discrepancies, after which the reviewers were retrained to standardize the evaluation criteria. The reviewers conducted a second independent assessment before the formal evaluation, achieving an ICC score of 0.984. During the formal evaluation, the reviewers assessed the sample content independently over one week. Differences were summarized and discussed, followed by a second assessment to ensure consistency. Any unresolved discrepancies were referred to a senior reviewer for final judgment.

### 2.4 Statistical analysis

All statistical analyses were conducted using SPSS for Windows (version 26.0; IBM Corp). The Shapiro-Wilk test examined whether data conformed to a normal distribution, and the median and the interquartile range (IQR) were used for descriptive analysis. For pairwise comparisons between multiple groups, the Kruskal-Wallis test and Bonferroni adjustment were performed. The Mann-Whitney test was used for independent two-sample comparisons, with Bilibili and Douyin as independent variables and the scores of the four evaluation criteria (DISCERN, JAMA, HRS, and GQS) as dependent variables. *P* < 0.05 was considered statistically significant.

## 3 Results

### 3.1 General features of websites and videos

In total, 177 websites and 460 videos were selected for inclusion in this study (**Figure 1**). In terms of search results presentation, an intriguing pattern emerged. On the Bing platform, we discovered that the first four and last four results on each page consistently comprised duplicate commercials. For specific search terms such as depression treatment, depressive anxiety, and depression on the Baidu platform, the first three results on each page were commercials. On the Douyin platform, search terms such as depression and depressive disorder initially yielded text-based depression content, followed by video content.

A total of 226 video authors were identified on the Douyin platform, and 66 on the Bilibili platform regarding identification. Most videos on both platforms were uploaded within the last two years. Of the 101 websites analyzed, the upload dates for 45 websites were unclear. Among the remaining websites, the upload dates ranged from 2009 to 2023, mostly uploaded after 2020 and including 37 uploaded in 2023.

**Figure 2:**
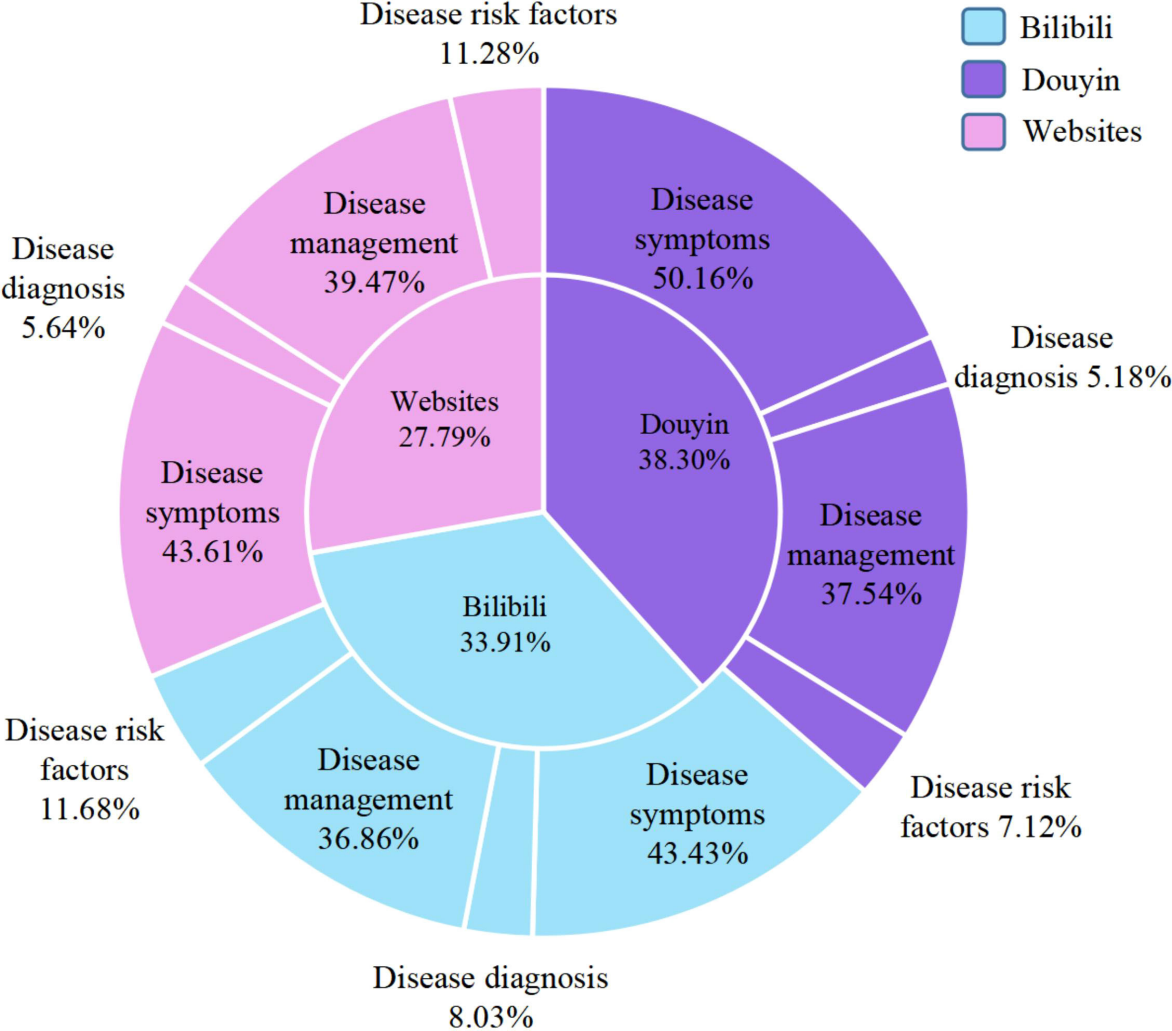
The percentage of each platform and its content category.

In terms of the classification percentage of contents and platforms, the Douyin platform accounted for the largest proportion among different platforms, comprising 38.30% of the total. Across all platforms, disease symptoms and disease management presented the dominant categories, while disease diagnosis represented the smallest proportion (**Figure 2**).

Regarding video-related metrics, the average duration of Bilibili was 172.00 seconds and Douyin was 48.00 seconds. Additionally, Douyin exhibited a higher average number of likes, comments, favorites, and shares compared to Bilibili (**Supplementary Table 5**).

### 3.2 Scoring analysis of websites and videos

Although websites obtained the highest DISCERN and HRS scores, with median scores (IQR) of 33 (29.00, 40.00) and 2 (1.50, 4.50) respectively, the grades based on the scores were determined to be “poor” and “very poor”. Bilibili, Douyin, and websites provided content of moderate quality (≥2 points) on JAMA, while their content quality on GQS was considered poor (< 3 points), with all median scores at 2 points (See **Supplementary Table 5**, which showed all descriptive statistics for all quality metrics).

Among these scales, there was no consensus on what scores signified good or poor quality when comparing different scales. Therefore, we converted these scores to percentages based on weights for cross-scale comparisons. The results indicated that only JAMA’s median (IQR) was more than half of the scores across all platforms, scoring 75.00 (75.00, 75.00) on Bilibili and 50.00 (50.00, 75.00) on Douyin and websites, respectively, while the other scales did not reach half of the scores (*P* < 0.001) (**Table 1**).

**Table 1:** Comparison of scores for videos and websites^a^.

	Bilibili	Douyin	Websites	*P* ^b^
	Median (IQR)	Median (IQR)	Median (IQR)	
DISCERN	37.50 (32.50, 41.25)	41.25 (36.25, 43.75)	41.25 (36.25, 50.00)	<.001
JAMA	75.00 (75.00, 75.00)	50.00 (50.00, 75.00)	50.00 (50.00, 75.00)	<.001
HRS	4.17 (4.17, 12.50)	8.33 (4.17, 12.50)	16.67 (12.50, 37.50)	<.001
GQS	40.00 (20.00, 45.00)	40.00 (40.00, 45.00)	40.00 (40.00, 60.00)	<.001

^a^All quality values were calculated using percentages.

^b^Calculated using the Kruskal-Wallis test.

### 3.3 Analysis of the different factors of video and website platforms

**Figure 3:**
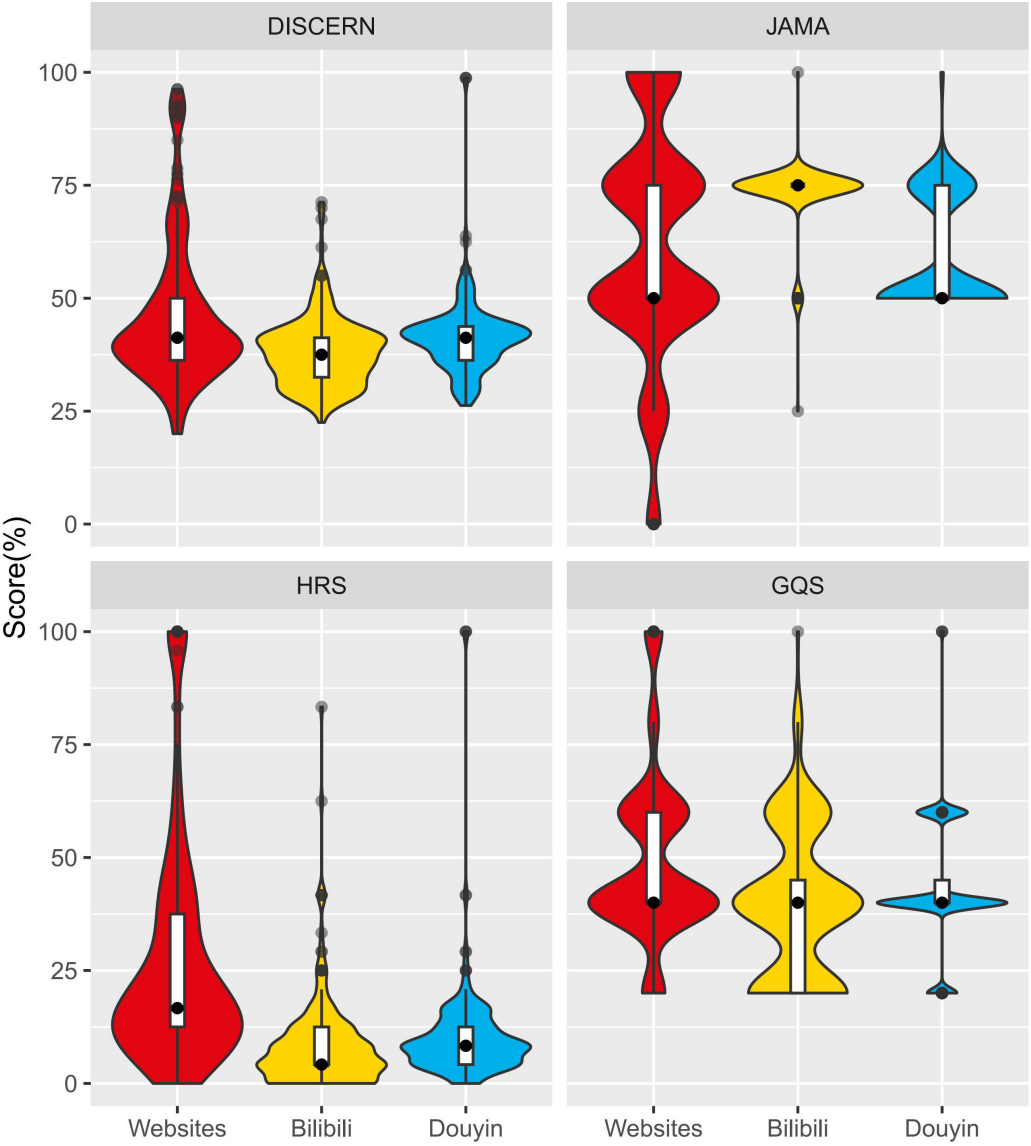
Comparison of the distribution of each quality score by platforms.

In this section of our study, we focused on comparing quality scores and video source categories for video platforms. To compare the quality scores of Bilibili and Douyin, we utilized the independent sample Mann-Whitney U test, revealing significant differences between the two platforms (*P* < 0.001). With the exception of JAMA, the scores of Douyin exceeded those of Bilibili in all other scales (**Figure 3**).

Additionally, the results using Kruskal-Wallis tests showed statistically significant differences between video sources and related metrics such as quality scores, number of comments (*P* = 0.001), number of likes (*P* = 0.002), and VPI (*P* < 0.001) (**Table 2**). Further Pairwise comparisons attributed these differences to the medical and non-medical categories (**Supplementary Table 6**), with medical organizations scoring highest on all four evaluation scales, followed by doctors and then news media with the lowest scores. Regarding video metrics, the non-medical professional categories held the longest average duration (*P* < 0.001), and the news media categories had the highest VPI among all categories (*P* < 0.001) (**Table 2**).

**Table 2:** Comparison of video source classifications with quality scores and related metrics.

	Doctors	Non-medical individuals	News media	Medical organizations	*P* ^a^
DISCERN					<.001
Median (IQR)	41.25 (40.00, 43.75)	36.25 (31.25, 41.25)	35.62 (29.69, 40.94)	43.75 (39.38, 51.56)	
JAMA					<.001
Median (IQR)	75.00 (50.00, 75.00)	75.00 (50.00, 75.00)	50.00 (50.00, 75.00)	75.00 (56.25, 75.00)	
HRS					<.001
Median (IQR)	8.33 (4.17, 12.50)	4.17 (2.08, 12.50)	4.17 (3.12, 8.33)	12.50 (8.33, 38.54)	
GQS					<.001
Median (IQR)	40.00 (40.00, 60.00)	40.00 (20.00, 40.00)	20.00 (20.00, 40.00)	40.00 (40.00, 60.00)	
Duration (seconds)					<.001
Median (IQR)	45.00 (26.50, 74.50)	183.00 (97.50, 403.50)	69.50 (34.00, 157.25)	59.50 (0.00, 78.50)	
Number of likes					<.001
Median (IQR)	2624.00 (609.50, 18500.00)	3478.00 (466.00, 17000.00)	54500.00 (16250.00, 182500.00)	1799.00 (272.00, 41000.00)	
Number of comments					0.002
Median (IQR)	290.00 (72.00, 2014.00)	435.00 (87.50, 1753.00)	3601.50 (866.25, 12000.00)	135.00 (33.00, 1948.00)	
Number of Favorites					0.001
Median (IQR)	725.00 (209.00, 3089.00)	1341.00 (237.50, 5540.00)	4071.00 (2082.00, 8710.00)	1831.00 (74.00, 4091.00)	
Number of shares					<.001
Median (IQR)	725.00 (111.00, 4249.50)	462.00 (58.00, 2884.50)	8000.00 (2923.75, 12750.00)	897.00 (111.00, 1849.00)	
VPI					<.001
Median (IQR)	0.03 (0.01, 0.17)	0.10 (0.02, 0.39)	0.46 (0.23, 1.17)	0.00 (0.00, 0.19)	

^a^Calculated using the Kruskal-Wallis test.

For websites, we used the Kruskal-Wallis test to analyze the relationship between its sources and scores, which showed news media categories and other categories both had the highest scores, while commercials had the lowest scores (**Supplementary Table 7**).

### 3.4 Websites platform versus video platform

When comparing websites to videos, we focused on assessing two aspects: the overall quality scores for all scales and the specific section scores for DISCERN and HRS. The findings revealed that websites scored higher in DISCERN, HRS, and GQS compared to Bilibili and Douyin (*P* < 0.001). Nevertheless, the JAMA score for websites was slightly lower than that of Bilibili and slightly higher than that of Douyin (**Figure 3**, **Table 1**).

**Figure 4:**
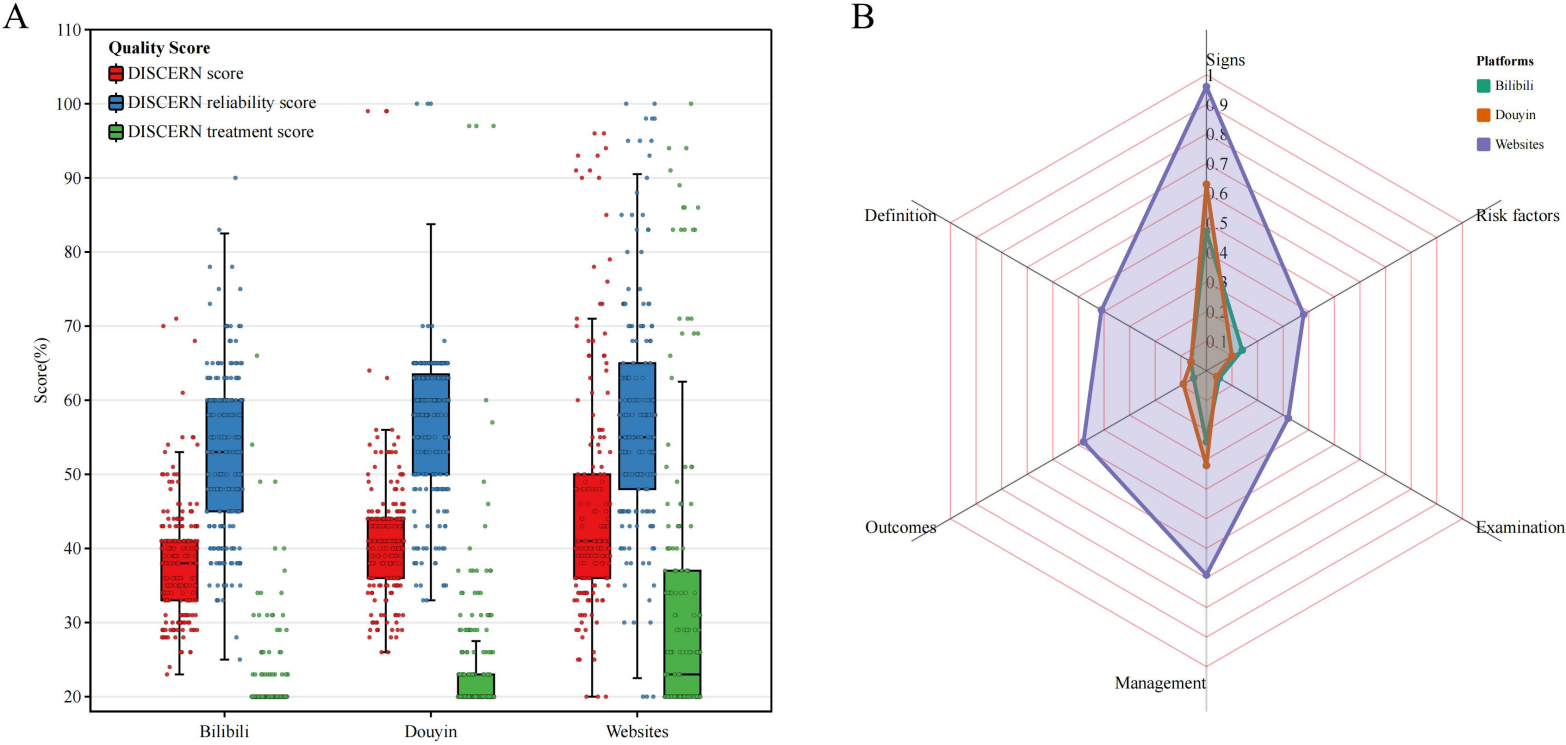
The scores of the different platforms in each section of the DISCERN **(A)** and HRS **(B)** tools.

Of the three different sections of the DISCERN tool, all platforms obtained relatively low scores for treatment and relatively high scores for reliability. The overall score and treatment score for websites outperformed those of Bilibili and Douyin; however, Douyin scored higher than websites in terms of reliability. As for Douyin, its scores surpassed those of Bilibili in all three sections (**Figure 4A**).

In terms of HRS scores across the six dimensions, websites outperformed videos on all dimensions, displaying the highest scores in symptom and management, while having the lowest score in examination on all platforms. Moreover, Douyin slightly outperformed Bilibili in symptoms, outcomes, and management (**Figure 4B**).

## 4 Discussion

### 4.1 Comparison with prior work

To the best of our knowledge, previous studies have focused on evaluating the information quality of websites across various health topics, including depression. However, there has been a lack of research specifically targeting Chinese depression-related information. Additionally, while some studies have assessed the quality of health information in general, none have specifically examined the quality of depression-related information on video platforms and compared it with websites, with the exception of a study comparing information on cosmetic injectables available on YouTube and websites (26). Therefore, this study aims to fill this gap by evaluating the quality of Chinese depression-related information online and comparing the information quality between websites and videos.

### 4.2 Principal results

Overall, our findings regarding depression-related content align with previous studies that have highlighted poor information quality available on both websites and videos (21, 22, 36). Websites demonstrated better quality when compared to videos, with Douyin displaying higher quality than Bilibili.

Among all content categories, disease symptoms and management constituted the highest proportion on each platform. News media exhibited the highest quality, while commercials had the lowest quality among all sources on websites. On video platforms, medical organizations and doctors had higher quality compared to other sources. Furthermore, search results on these platforms tended to display recent information, and depression-related website recommendations often featured advertisements, whereas video platforms did not present ad-containing results.

#### 4.2.1 The difference in quality across platforms

Our study revealed that videos, as identified by different search engines, exhibited lower quality compared to websites, which is consistent with previous research (26). This difference in quality could be attributed, in part, to the constraints of video length and viewer engagement, which may limit content diversity. In contrast, websites can provide more detailed information.

Online platforms primarily presented information on depressive disorders from a Western medicine perspective, with significantly less content related to Traditional Chinese Medicine (TCM). Websites generally provided more comprehensive and higher-quality TCM information than video platforms, suggesting that websites remain the primary communication channel for users seeking TCM-related advice and services (37). However, online TCM-related health information on depressive disorders remained limited. This limitation may stem from a greater emphasis on public education and research concerning Western medicine for depressive disorders, leading people to perceive Western medicine as the mainstream treatment and resulting in an abundance of Western medicine-focused content online (38).

When examining video quality, it was observed that Bilibili had overall inferior quality compared to Douyin. This discrepancy may arise due to differences in audience demographics and the ratio of unidentified authors in depression-related videos on Bilibili as compared to Douyin.

Concerning video-related metrics and quality, we observed no correlation between duration and quality scores, differing from previous studies (37, 39). However, the relationship between likes, comments, favorites, and shares and the quality score aligns with prior findings: the higher the number of these metrics, the lower the quality score (25, 39). This pattern suggests that video platforms would prioritize popularity and viewer preference to present videos rather than considering the quality of depression-related videos (40).

#### 4.2.2 Content categories and sources for different platforms

Regarding content categories on websites and videos, both Disease Symptoms and Disease Management accounted for the highest proportion on each platform. In general, websites demonstrated higher quality scores compared to videos, with Douyin exhibiting higher quality scores than Bilibili. These results imply that the majority of online depression information focuses on disease symptoms and coping strategies. Consequently, patients may need to seek further in-person consultations to confirm specific screenings and disease outcomes (26). The role of online health information serves the purpose of providing individuals with foundational knowledge about the disease, such as symptoms and management, enabling viewers to make initial assessments based on their own situations and determine if further medical assistance is necessary.

The quality of news media was the best but the quality of commercials was the worst among all sources on websites, which is consistent with previous research findings (22, 41). This implies that commercial websites prioritize advertising effectiveness over the quality of information when sharing depression-related content with their audience. On the other hand, news media websites prioritize objective information and focus on the quality of depression-related content. When considering video sources, although news media and non-medical professionals tend to have longer videos than doctors and medical organizations, the latter showcase better quality across all video sources.

#### 4.2.3 Results presented on different platforms

Regarding the display of search results on websites and videos, it was observed that these platforms tend to provide recent results, but little improvement in quality was noted compared to previous studies (11, 21, 42). Furthermore, recommendation algorithms for depression-related information websites often feature advertisements that are of poor quality. In contrast, video platforms did not present any ad-containing results. Notably, on Douyin, when searching for “depression” or “depressive disorder,” a doctor-vetted information page precedes relevant video content. This aligns with the recommendation from previous research to ensure viewers have access to authoritative, accurate, and high-quality health information (25). However, such a feature was not present on the Bilibili platform.

### 4.3 Limitations

This study has several limitations. Firstly, the data collected may not be representative of different time periods and individuals, as online information is continuously updated and search results can be influenced by factors such as video recommendation algorithms, IP addresses, and devices. Additionally, this study focused on the most popular platforms and commonly used search terms, potentially overlooking other platforms and individual preferences, resulting in a lack of representation of certain information sources. Lastly, because the characteristics of videos and websites are different, conducting comparisons in terms of views, likes, and shares is limited. This constraint may affect the interpretation of additional information.

### 4.4 Conclusion

The growth of the internet has significantly changed the way we access online information, with a shift from traditional websites to popular videos. This study evaluated and compared the quality of Chinese depression-related information on websites and videos. The results revealed that, overall, the quality of depression-related information available on the internet was poor. Websites exhibited higher quality compared to videos, with Douyin displaying better quality than Bilibili. Notably, news media sources generally provided higher quality content on websites, while medical organizations and doctors showcased higher quality on video platforms. These results highlight the importance of collaborative efforts between platforms and professionals to improve the quality of depression-related information. Additionally, there is a need to prioritize algorithmic recommendations based on information quality rather than relying solely on popularity when presenting search results.

## Data Availability

The raw data supporting the conclusions of this article will be made available by the authors, without undue reservation.
