# Supplementary material for: Evaluation of the quality of depression-related information on Chinese websites and video platforms: a cross-sectional comparative analysis
Source: Front Psychiatry. 2024 Dec 12;15:1408384. doi: 10.3389/fpsyt.2024.1408384 (PMC11669601; doi:10.3389/fpsyt.2024.1408384)
Supplement: Supplementary file 1 [file Table1.docx]

|  |  | Question | Score |
| --- | --- | --- | --- |
| Section 1 | 1 | Are the aims clear? | 1 to 5 |
|  | 2 | Does it achieve its aims? | 1 to 5 |
|  | 3 | Is it relevant? | 1 to 5 |
|  | 4 | Is it clear what sources of information were used to compile the publication (other than the author or producer)? | 1 to 5 |
|  | 5 | Is it clear when the information used or reported in the publication was produced? | 1 to 5 |
|  | 6 | Is it balanced and unbiased? | 1 to 5 |
|  | 7 | Does it provide details of additional sources of support and information? | 1 to 5 |
|  | 8 | Does it refer to areas of uncertainty? | 1 to 5 |
|  |  |  |  |
| Section 2 | 9 | Does it describe how each treatment works? | 1 to 5 |
|  | 10 | Does it describe the benefits of each treatment? | 1 to 5 |
|  | 11 | Does it describe the risks of each treatment? | 1 to 5 |
|  | 12 | Does it describe what would happen if no treatment is used? | 1 to 5 |
|  | 13 | Does it describe how the treatment choices affect overall quality of life? | 1 to 5 |
|  | 14 | Is it clear that there may be more than 1 possible treatment choice? | 1 to 5 |
|  | 15 | Does it provide support for shared decision making? | 1 to 5 |
|  |  |  |  |
| Section 3 | 16 | Based on the answers to all of these questions, rate the overall quality of the publication as a source of information about treatment choices. | 1 to 5 |

**Supplementary Table 1. DISCERN scoring system.**

**Supplementary Table 2. JAMA** **Benchmark Criteria.**

| JAMA Benchmarks | Explanation | Score |
| --- | --- | --- |
| Authorship | Authors and contributors, their affiliations, and relevant credentials should be provided | 0 or 1 |
| Attribution | References and sources for all content should be listed clearly, and all relevant copyright information should be noted | 0 or 1 |
| Disclosure | Website “ownership” should be prominently and fully disclosed, as should any sponsorship, advertising, underwriting, commercial funding arrangements or support, or potential conflicts of interest | 0 or 1 |
| Currency | Dates when content was posted and updated should be indicated | 0 or 1 |

**Supplementary Table 3. Hexagonal Radar Schema**

| Category | Criteria | Score |
| --- | --- | --- |
| Definition | Defining a disease or a particular stage, type, category of the disease | Not addressed at all = 0 Slightly addressed = 0.5 Partially addressed = 1 Quite well addressed = 1.5 Fully addressed = 2 |
| Signs | Typical signs caused by the disease |  |
| Risk factors | Factors that might cause the incidence of the disease or accelerate its progress |  |
| Examination | Means used for diagnosing and evaluating the disease |  |
| Management | Treatment |  |
| Outcomes | Prognosis of the disease, complications, survival or death |  |

**Supplementary Table 4. Description of the Global Quality Score 5-point scale**

| Description | Score |
| --- | --- |
| Poor quality; poor flow of the site; most information missing; not at all useful for patients | 1 |
| Generally poor quality and poor flow; some information listed but many important topics missing; of very limited use to patients | 2 |
| Moderate quality; suboptimal flow; some important information is adequately discussed but others poorly discussed; somewhat useful for patients | 3 |
| Good quality and generally good flow; most of the relevant information is listed, but some topics not covered; useful for patients | 4 |
| Excellent quality and excellent flow; very useful for patients | 5 |
